# TGF-β and Iron Differently Alter HBV Replication in Human Hepatocytes through TGF-β/BMP Signaling and Cellular MicroRNA Expression

**DOI:** 10.1371/journal.pone.0039276

**Published:** 2012-06-18

**Authors:** Sun O. Park, Mukesh Kumar, Sanjeev Gupta

**Affiliations:** 1 Department of Medicine, Albert Einstein College of Medicine of Yeshiva University, Bronx, New York, United States of America; 2 Department of Pathology, Albert Einstein College of Medicine of Yeshiva University, Bronx, New York, United States of America; 3 Marion Bessin Liver Research Center, Cancer Center, Diabetes Research Center, Ruth L. and David S. Gottesman Institute for Stem Cell and Regenerative Medicine Research, and Institute for Clinical and Translational Research, Bronx, New York, United States of America; Drexel University College of Medicine, United States of America

## Abstract

The nature of host-virus interactions in hepatitis B virus infection is incompletely understood. Since soluble factors, e.g., cytokines and metals, may exacerbate liver injury in chronic hepatitis, we considered that defining the effects of receptor-mediated signaling upon viral replication will be significant. Consequently, we studied effects of iron or TGF-β-induced TGF-β/BMP signaling in the HepG2 2.2.15 cell model of hepatitis B virus replication. We found iron and TGF-β increased hepcidin mRNA expression or TGF-β receptor kinase activity, respectively, which indicated that 2.2.15 cells responded appropriately to these substances. However, iron increased but TGF-β decreased hepatitis B virus mRNA and DNA expression. TGF-β induced expression at the mRNA level of multiple TGF-β/BMP pathway genes. This change was not observed in iron-treated cells. On the other hand, presence of SMAD proteins in iron or TGF-β-treated cells, including of SMAD4, did confirm convergence of TGF-β/BMP signaling pathways under these conditions. Since transcription factors in TGF-β/BMP signaling pathways could not have directly targeted hepatitis B virus itself, we studied whether iron or TGF-β exerted their effects through alternative mechanisms, such as by involvement of antiviral cellular microRNAs. We discovered cellular microRNA expression profiles were significantly different in iron or TGF-β-treated cells compared with untreated control cells. In many cases, exposure to iron or TGF-β changed microRNA expression in opposite directions. Introduction in cells of sequences representing such differentially expressed microRNAs, e.g., hsa-miR-125a-5p and -151-5p, even reproduced effects on virus replication of iron- or TGF-β. We surmised that TGF-β/BMP pathway members, i.e., SMADs, likely governed iron or TGF-β-induced microRNA expression. Iron may have mediated Drosha/DGCR8/heme-mediated processing of microRNAs. In turn, cellular microRNAs regulated replication of hepatitis B virus in iron or TGF-β-treated cells. This knowledge should advance studies of mechanisms in viral-host interactions, hepatic injury, and therapeutic developments for hepatitis B.

## Introduction

Intra- and extracellular soluble signaling molecules are involved in hepatitis virus replication but these interactions are not well understood. For instance, inflammatory cytokines affect hepatitis B virus (HBV) replication by recruiting more than one signaling pathways. Among these, interleukins (e.g., IL12, IL18) may inhibit HBV replication, including with recruitment of interferon (IFN)-γ released from NK or T cells [1], [2]. Interferon-α has widely been used for treating HBV with JAK/STAT signaling serving intermediary roles [3]. The role of these intracellular signaling pathways in transducing antiviral effects of interferon is far from complete and new information is still emerging [4]. Other cytokine pathways off note include tumor necrosis factor-α, which suppressed HBV replication [5]. Also, transforming growth factor (TGF)-β inhibited HBV replication [6], presumably with involvement of TGF-β signaling through SMAD-2 and -3 [7]. The canonical TGF-β signaling pathways involve SMADs -2 and -3 compared with bone morphogenetic protein (BMP) signaling via SMAD-1, -5, and -8. Nonetheless, after activation of TGF-β- or BMP receptors leads to heteromeric complexing between SMADs, followed by engagement with the common-mediator, SMAD-4, which is required and sufficient for regulation of nuclear transcription, and in this way, brings together TGF-β/BMP signaling pathways. How these diverse intracellular signaling pathways may regulate replication of HBV (or other viruses) is yet to be clarified.

Disease-modifying cofactors, e.g., iron, are capable of altering HBV replication. In clinical studies, elevated hepatic iron content has been associated with higher prevalence of HBV infection [8], as well as worse outcomes in chronic hepatitis [9]. However, the molecular basis by which iron may alter HBV replication is unknown. Recently, hepatic release of hepcidin was found to be important in iron homeostasis, by decreasing intestinal iron absorption as well as hepatic iron uptake. As hepcidin exerts its intracellular effects by TGF-β/BMP signaling [10], a relationship emerged between this molecule and other intracellular mediators of cytokines. Unexpected signaling mechanisms were found to regulate hepcidin expression, e.g., epidermal growth factor, and also hepatocyte growth factor, which transduced their effects on hepcidin through PI3 kinase or MEK/ERK pathways [11]. Interestingly, iron-induced hepcidin expression altered HCV replication in cultured cells [12].

Therefore, intracellular signaling pathways could regulate hepatitis virus replication in many ways. More knowledge in this area will be significant for virus-host interactions and hepatic injury. Also, cytokines, chemokines and receptors expressed during host-viral interactions have elicited interest as targets for antiviral therapies [13].

Here, we reasoned that study of intersections in signaling by cytokines, e.g., TGF-β, on the one hand, and signaling by small molecules, e.g., iron, on the other hand, would help clarify mechanisms in HBV replication. One possibility was that iron and TGF-β exerted their effects by mechanisms completely independent of one another. Another possibility was that these molecules shared common intermediaries but that were regulated differently.

We used HepG2 2.2.15 cells for our studies since these express stably transduced HBV genomes with virus replication and have been suitable for mechanistic studies in vitro [14], [15]. This cell line model permitted us to characterize the effects of TGF-β and iron on HBV replication, to demonstrate activation of intracellular signaling along TGF-β and BMP pathways, and to establish potential effector mechanisms in regulation of HBV replication.

## Materials and Methods

### Cytokines and Chemicals

We purchased TGF-β (R&D Systems, Minneapolis, MN) and ferrous sulfate heptahydrate (Sigma Chemical Co., St. Louis, MO). TGF-β1 receptor kinase inhibitor (TKI), LY-2157299 (Eli Lilly and Co., Indianapolis, IN) was from Dr. A. Verma at Einstein. All reagents and chemicals were from Sigma.

### Cells

HepG2 2.2.15 cells were derived from HepG2 cells stably transfected with full-length HBV genomes (14). HepG2 cells were originally from American Type Culture Collection (ATCC, Manassas, VA). Cells were cultured in Dulbecco’s Modified Eagle’s medium with 10% fetal bovine serum, 1% glutamine, 1% nonessential amino acids, and antibiotics (DMEM). After dose-ranging studies, we used 20 ng/ml TGF-β, 100 µM iron or 480 ng/ml TKI with cell culture for up to 48 h. For assays, cells were lysed in 100 mM Tris-HCl, pH 8.0, 0.2% Nonidet P-40 and protease inhibitors (Calbiochem Cocktail set III, EMD4Biosciences, Darmstadt, Germany). Protein content was measured by Bradford Reagent (Pierce Research Products, Rockford, IL). To evaluate cell viability or changes in cell numbers, MTT assays were performed, as described previously [15].

### HBV Assays

To demonstrate hepatitis B core antigen (HBcAg), cell lysates were resolved in 1.2% native agarose gels, followed by transfer to nylon membranes (Ambion, Life Technologies, Carlsbad, CA). HBcAg was detected with anti-HBc (Dako, Carpinteria, CA), as described previously [14]. For southern blotting, protein-detergent complexes were precipitated in 2.5 M KCl, viral pellet was dissolved in Tris-EDTA, and incubated with proteinase K (Ambion) for 1 h at 37°C. DNA was extracted with phenol-chloroform and precipitated by ethanol. 20 µg DNAs were resolved in 1.2% agarose gels and transferred to nylon membranes (Ambion). To analyze HBV RNA with northern blotting, 20 µg total cellular RNAs were extracted by Trizol Reagent (Life Technologies), resolved in 1.0% formaldehyde-agarose, and transferred to nylon membranes (Ambion). Nonradioactive HBV probe was prepared with psoralen-Biotin labeling kit (Ambion), as described by the manufacturer, with full-length HBV DNA from EcoRI- linearized pCP10 plasmid [16]. Prehybridization and hybridization of blots was as described previously [15]. Northern blots were rehybridized with human glyceraldehyde-3-phosphate dehydrogenase (GAPDH) probe, as described previously [15]. Replicate blots were quantified by densitometry.

### TGF-β Receptor Kinase Activity

Cell lysates with 50 µg total proteins each were spotted onto nylon membranes (Ambion). Blots were probed with mouse anti-phospho-serine-threonine antibody (Cat#612548, BD Biosciences, San Diego, CA). Detection used anti-mouse IgG-peroxidase (Sigma) with chemiluminiscence methods.

### Cellular Gene Expression

Total cellular RNAs were isolated with TRIzol Reagent (Life Technologies). Hepcidin expression was analyzed by quantitative real-time (RT)-polymerase chain reactions (PCR) with SYBR Green master mix (Qiagen, Valencia, CA). cDNAs were prepared with Omniscript RT kit (Qiagen). Hepcidin primers were: sense 5′-CTGCAACCCCAGGACAGAG-3′ and antisense 5′-GGAATAAATAAGGAAGGGAGGGG-3′ [17]; GAPDH primers were: sense 5′-GGCCTCCAAGGAGTAAGACC-3′, and antisense 5′-AGGGGTCTACATGGCAACTG-3′. PCR conditions were: 40 cycles of denaturation at 95°C×90 s, annealing at 60°C×10 s and elongation at 72°C×20 s. For TGF-β/BMP signaling pathways, an array with 84 genes was used for qRT-PCR (http://www.sabiosciences.com/rt_pcr_product/HTML/PAHS-035A.html, SA Biosciences, Gaithersburg, MD). cDNAs were prepared by RT^2^ First Strand Kit. Each condition was in triplicate. Relative gene expression was determined by 2∧(-ΔΔCt) method.

### Immunostaining

Cells were fixed in methanol at −20°C. Receptor-activated phosphorylated forms of R-SMAD-2, -3 and -4 were stained with antibodies (Cat#3101, #9515, #9520, Cell Signaling, Danvers, MA), followed by detection with Alexa Fluor 633-conjugated goat anti-rabbit IgG (Molecular Probes, Life Technologies). Nuclei were counterstained by DAPI.

**Figure 1:**
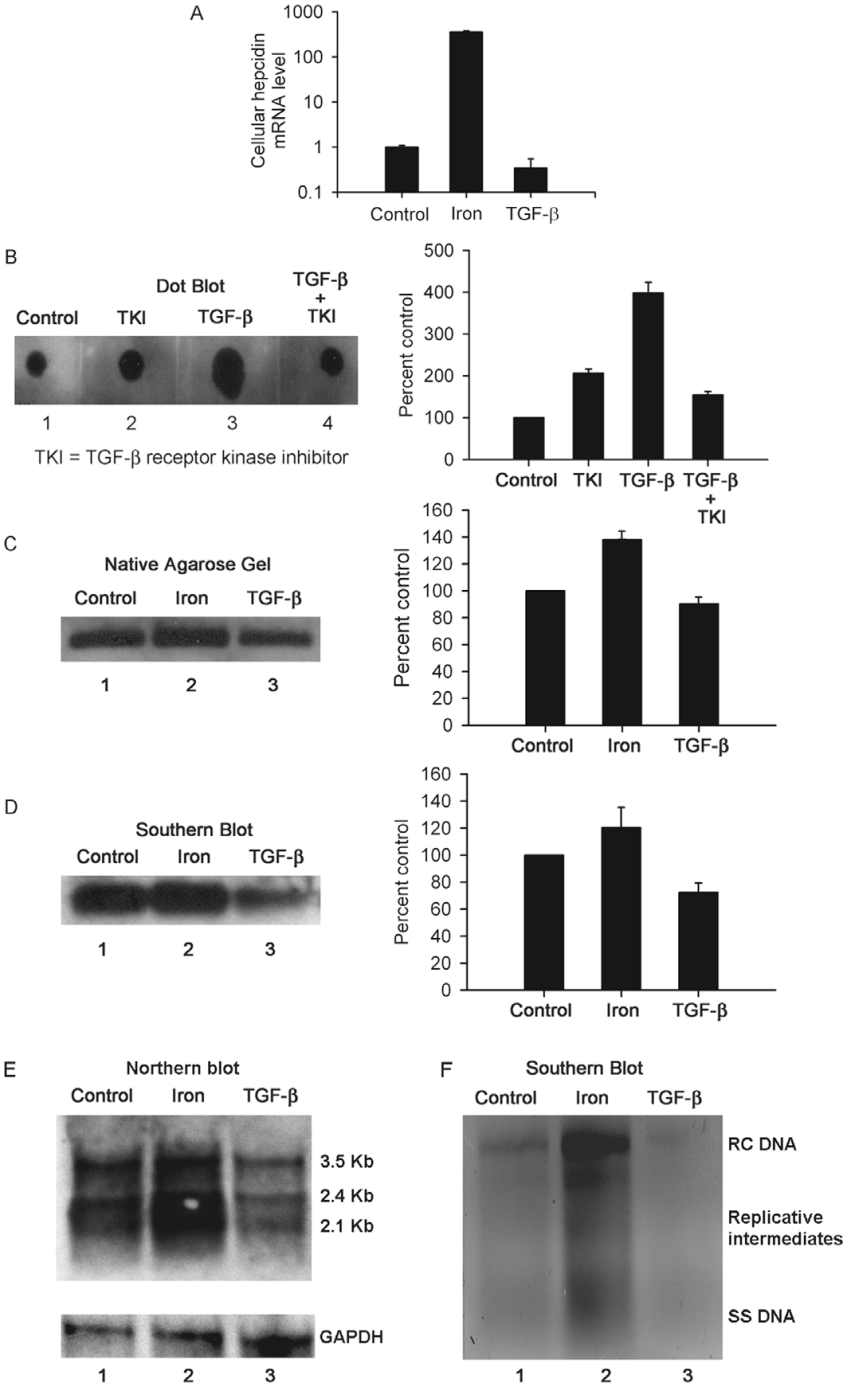
Effects of TGF-β and iron on 2.2.15 cells. Shown are studies with cells cultured in the presence of 20 ng/ml TGF-β or 100 µM iron for 48 h. (A) Hepcidin mRNA expression increased by several-fold in iron-treated cells and not in TGF-β-treated cells. These data are plotted on a natural log scale in comparison with controls. (B) Serine-threonine kinase activity in cells with increase after TGF-β. This increase in kinase activity was blocked in cells treated simultaneously with TKI. The chart on the right shows results of densitometric scanning in replicates. (C) Native agarose gel showing HBcAg expression, along with densitometric scanning of bands on right. Iron increased and TGF-β decreased HBV replication. (D) Southern blot of native agarose gel confirming changes in HBcAg-associated HBV DNA with chart on right showing densitometric quantitation of bands. (E) Northern blot of HBV mRNA expression with 3.5, 2.4 and 2.1 Kb HBV transcripts as indicated. (F) Southern blot of DNA from cell lysates showing relaxed-circular (RC), single-stranded (SS) and intermediate replicative forms of HBV DNA. Taken together, these results confirmed that cells were responsive to iron and TGF-β with the former increasing and the latter decreasing HBV replication.

### MicroRNA Studies

Profiles of microRNA expression were obtained in total cellular RNA samples by microfluidics approach with arrays (Sanger databases, versions 9.1 and 11.0; LC Sciences, Dallas, TX). Background was subtracted with regression-based mapping on 5–25% of lowest intensity data excluding blank spots. Transcripts with undetectable signals below 3× background, incorporating spot analysis parameters, were excluded. Later, we excluded transcripts with <500 arbitrary signals as these are not identified by qRT-PCR. RNA hybrids were examined in HBV subtype ayw (Genbank accession number ×65257.1) with miRanda program using minimal free energy (mfe) below −12 kcal/mol and miRanda score of over 140 as selection criteria.

Oligonucleotide mimics or antagonists of microRNA were from commercial source (Dharmacon, Thermo Fisher): 125-a-5p, UCCCUGAGACCCUUUAACCUGUGA; 151-5p, UCGAGGAGCUCACAGUCUAGU. For transfections, cells were incubated for 6 h with 50–200 nM of oligonucleotides in lipofectamine (Invitrogen). Transfection efficiency was monitored with 100 nM of Silencer Cy3-labeled Negative Control #1 siRNA (AM4621, Ambion). Iron or TGF-β were added to cells 12 h after miRNA transfections. Cells were cultured with Iron or TGF-β for 48 h before analysis of HBV replication.

### Statistical Methods

Data are shown as means ± standard errors. T-tests or analysis of variance (ANOVA) with Turkey’s multiple comparisons test were used with Prism 5 (GraphPAD 5.0, San Diego, CA). P values <0.05 were taken as significant. Critical studies were repeated 2–4 times.

**Figure 2:**
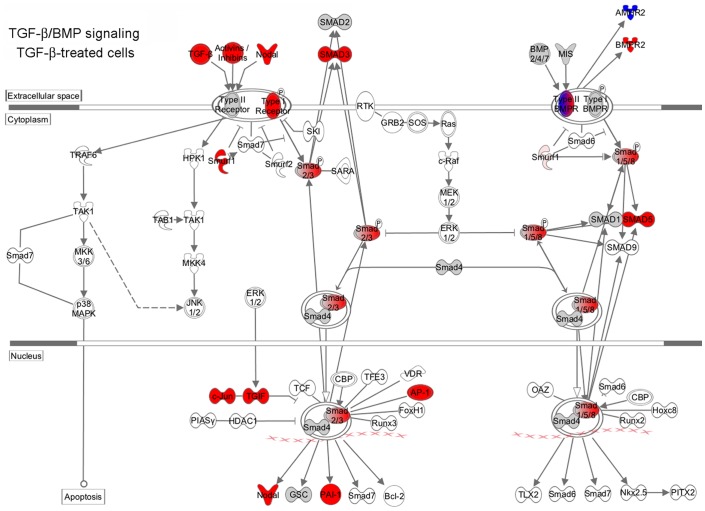
Mapping of changes in TGF-β/BMP signaling. Shows gene expression changes in 2.2.15 cells treated by 20 ng/ml TGF-β for 48 h versus untreated controls. Changes were mapped with curated pathways in Ingenuity Pathway Analysis. Genes with increased expression are shown in red color and genes with decreased expression are shown in blue relative to untreated control cells.

**Table 1:** Fold increase in TGF-β/BMP pathway gene expression versus untreated cells (NC = no change).

Gene Groups	Gene	Gene name	Iron	TGF-β
**TGF-β Superfamily Cytokines**	LEFTY1	Left-right determination factor 1	NC	**339**
	NODAL	Nodal homolog (mouse)	NC	**40**
	LTBP2	Latent transforming growth factor beta binding protein 2	NC	**30**
	TGFB2	Transforming growth factor, beta 2	NC	**21**
	TGFB1	Transforming growth factor, beta 1	NC	**13**
	INHA	Inhibin, alpha	NC	**11**
	INHBA	Inhibin, beta A	NC	**5**
	BMP1	Bone morphogenetic protein 1	NC	**5**
	LTBP1	Latent transforming growth factor beta binding protein 1	NC	**4**
	INHBB	Inhibin, beta B	NC	**3**
	TGFB3	Transforming growth factor, beta 3	NC	**3**
**Receptors**	TGFB1I1	Transforming growth factor beta 1 induced transcript 1	NC	**10**
	BMPR2	Bone morphogenetic protein receptor, type II	NC	**8**
	ACVR1	Activin A receptor, type I	NC	**6**
	TGFBR1	Transforming growth factor, beta receptor 1	NC	**5**
	BMPR1A	Bone morphogenetic protein receptor, type IA	NC	**2**
**SMADs and Target Genes**	JUNB	Jun B proto-oncogene	NC	**43**
	PDGFB	Platelet-derived growth factor beta polypeptide	NC	**16**
	CDKN2B	Cyclin-dependent kinase inhibitor 2B (p15, inhibits CDK4)	NC	**26**
	COL1A1	Collagen, type I, alpha 1	NC	**14**
	ENG	Endoglin	NC	**12**
	IGF1	Insulin-like growth factor 1 (somatomedin C)	NC	**11**
	SERPINE1	Serpin peptidase inhibitor, clade E (nexin, plasminogen activator inhibitor type 1), member 1	NC	**11**
	FST	Follistatin	NC	**7**
	SOX4	SRY (sex determining region Y)-box 4	NC	**6**
	DLX2	Distal-less homeobox 2	NC	**6**
	TGFBI	Transforming growth factor, beta-induced, 68 kDa	NC	**6**
	BAMBI	BMP and activin membrane-bound inhibitor homolog	NC	**5**
	JUN	Jun proto-oncogene	NC	**5**
	RUNX1	Runt-related transcription factor 1	NC	**4**
	SMAD3	SMAD family member 3	NC	**4**
	CDKN1A	Cyclin-dependent kinase inhibitor 1A (p21, Cip1)	NC	**4**
	TGIF1	TGFB-induced factor homeobox 1	NC	**4**
	SMURF1	SMAD specific E3 ubiquitin protein ligase 1	NC	**3**
	FOS	FBJ murine osteosarcoma viral oncogene homolog	NC	**3**
	ID1	Inhibitor of DNA binding 1	NC	**3**
	ITGB5	Integrin, beta 5	NC	**2**
	NBL1	Neuroblastoma, suppression of tumorigenicity 1	NC	**2**
	SMAD5	SMAD family member 5	NC	**2**
	NBL1	Neuroblastoma, suppression of tumorigenicity 1	NC	**2**

**Figure 3:**
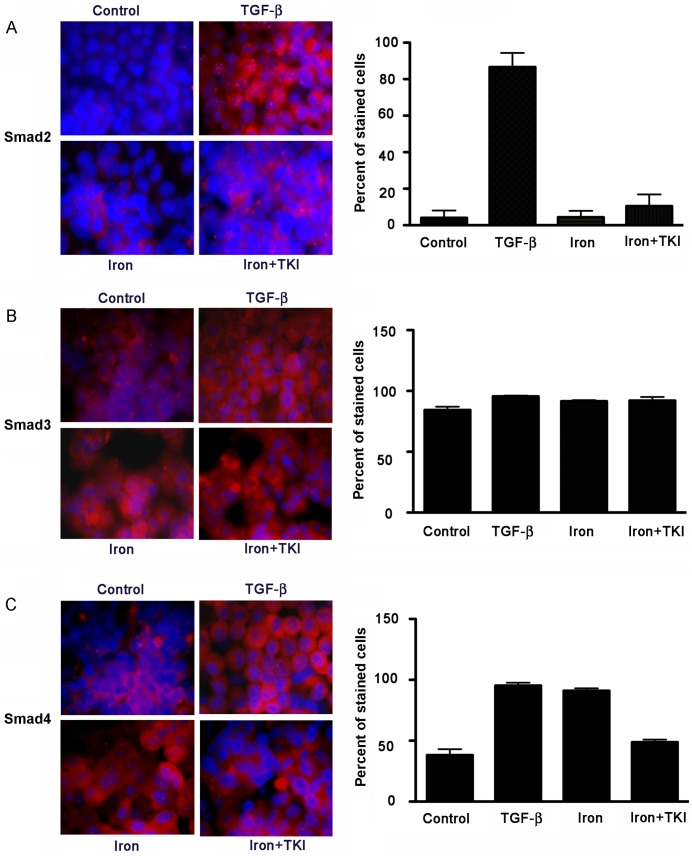
Regulation of SMAD expression in cells. Cells were cultured with 20 ng/ml TGF-β, 100 µM iron, or 100 µM iron plus 480 ng/ml TKI for 48 h. Immunostaining of cultured cells is shown in representative images for phosphorylated SMAD-2 (A), SMAD-3 (B) and SMAD-4 (C) (red color with DAPI counterstaining of nuclei in blue color). Morphometric analysis of larger samples of cells is given in accompanying charts. TGF-β increased expression of SMAD-2 and SMAD-4 but not SMAD-3. By contrast, iron had no effect on expression of SMAD-2 or SMAD-3. However, iron increased SMAD-4 expression, which was partly blocked by TKI. These results indicated differences in the nature of intracellular signaling in cells treated with iron or TGF-β although activation of SMAD-4 by both iron and TGF-β indicated confluence of TGF-β/BMP signaling in these conditions. This was emphasized by lowering of SMAD-4 expression in cells treated with iron plus TKI. Orig. mag., x630.

**Table 2:** Changes in microRNA expression versus control untreated cells.

Downregulated		Upregulated	
MicroRNA	Log2 fold-change	MicroRNA	Log2 fold-change
Iron-treated			
hsa-miR-106a	−8.38	hsa-let-7a	2.66
hsa-miR-1246	−6.80	hsa-let-7b	7.20
hsa-miR-125a-5p	−4.06	hsa-let-7c	6.33
hsa-miR-1275	−1.69	hsa-let-7d	3.6
hsa-miR-128	−2.74	hsa-let-7e	2.41
hsa-miR-148a	−7.64	hsa-let-7f	2.66
hsa-miR-151-3p	−1.79	hsa-let-7g	1.58
hsa-miR-151-5p	−2.06	hsa-let-7i	2.51
hsa-miR-17	−7.64	hsa-miR-1224-5p	4.17
hsa-miR-18a	−7.16	hsa-miR-1228*	5.51
hsa-miR-192	−4.64	hsa-miR-1268	2.49
hsa-miR-194	−3.09	hsa-miR-1305	3.93
hsa-miR-200b	−5.64	hsa-miR-130a	2.58
hsa-miR-20a	−10.70	hsa-miR-15b	1.14
hsa-miR-20b	−8.38	hsa-miR-182	1.38
hsa-miR-21	−2.18	hsa-miR-183	2.04
hsa-miR-224	−8.97	hsa-miR-188-5p	4.10
hsa-miR-26a	−1.79	hsa-miR-195	5.68
hsa-miR-320a	−2.64	hsa-miR-206	4.54
hsa-miR-320b	−2.18	hsa-miR-210	3.42
hsa-miR-320c	−2.40	hsa-miR-22	2.23
hsa-miR-320d	−2.47	hsa-miR-23a	1.07
hsa-miR-361-5p	−1.40	hsa-miR-29c	4.09
hsa-miR-423-5p	−1.40	hsa-miR-300	3.97
hsa-miR-454	−7.16	hsa-miR-30b	1.63
hsa-miR-455-3p	−5.06	hsa-miR-30c	2.20
hsa-miR-605	−5.64	hsa-miR-31	8.58
hsa-miR-638	−1.12	hsa-miR-34c-3p	4.43
hsa-miR-663	−3.47	hsa-miR-568	9.87
hsa-miR-720	−2.06	hsa-miR-574-3p	6.61
hsa-miR-877	−5.06	hsa-miR-574-5p	4.07
hsa-miR-92a	−5.06	hsa-miR-595	5.29
hsa-miR-92b	−9.97	hsa-miR-601	5.88
		hsa-miR-765	4.47
		hsa-miR-98	5.05
		hsa-miR-99a	6.41
**TGF-β-treated**			
hsa-miR-20b	−1.29	hsa-let-7a	1.38
hsa-miR-221	−1.25	hsa-let-7d	1.43
hsa-miR-605	−4.64	hsa-let-7e	2
hsa-miR-638	−1.40	hsa-miR-125a-5p	2.87
hsa-miR-663	−2.06	hsa-miR-146a	2.72
hsa-miR-720	−2.40	hsa-miR-21	1.14
		hsa-miR-23a	1.20
		hsa-miR-23b	1.14
		hsa-miR-30c	1.89
		hsa-miR-483-5p	1.38
		hsa-miR-574-5p	2.23
		hsa-miR-99b	1.63

**Table 3:** Properties of miRNA capable of targeting HBV.

miRNA	HBV target (nt)and (HBV gene)	Mfe(kcal/mol)	miRandascore	Sequence alignment
hsa-let-7a	90–111(P/S)	−18.6	153	**miRNA** **3′ UUGAUAUGUUGGAUGAUGGAGU 5′** **5′ CCCTGTT CCGACTACT GCCTCT 3′ target**
hsa-let-7d	91–111(P/S)	−19.3	151	**miRNA** **3′ UGAUACGUUGGAUGAUGGAGA 5′** **5′ CCTG TTCCGACTACTG CCTCT 3′ target**
	3075–3104(P/S)	−15.3	146	**5′ miRNA** **3′ UGAUACG–––––-UU––––GGAUGAUGGAGA** **5′ ACTTT GCCAGCAAATCCGCCTCCTGCCTCC 3′ target**
hsa-let-7e	3084–3104(P/S)	−20.4	154	**miRNA** **3′ UGAUAUGUUGGAGGAUGGAGU 5′** **5′ GCAAATCCGCC TCCTGCC TCC 3′ target**
	91–111(P/S)	−15.9	146	**miRNA** **3′ UGAUAUGUUGGAGGAUGGAGU 5′** **5′ CCTG TTCCGACTACTG CCTCT 3′ target**
hsa-miR-106a	1568–1590(P/X)	−19	151	**miRNA** **3′ CGAUGGAC–GUGACAUUCGUGAAAA 5′** **5′ TCT GCCTGACCTTGT––-GCACTTCG 3′ target**
hsa-miR-125a-5p	1451–1472(P/X)	−19.36	151	**miRNA** **3′ GUGUCCAAUUUCCCAGAGUCCCU 5′** **5′ CGC–GGACGACCCGTCTCGGGGT 3′ target**
	3038–3065 (P/S/X)	−18.4	161	**5′ miRNA** **3′ GUGUCCAA UUUC––––––-CCAGAGUCCCU** **5′ TTT GGGGTGGAGCCCTCAGG–CTCAGGGC 3′ target**
hsa-miR-148a	130–154(P/X)	−15.4	140	**miRNA** **3′ UGUUUCAAGACAUC––––-ACGUGACU 5′** **5′ TCGAGGAT–TGGGGACCCTGCGCTGA 3′ target**
hsa-miR-151	231–254(P/S)	−12.2	144	**5′miRNA** **3′ GGAGU–UCCU––CGAAGUCAGAUCA** **5′ CC TCACAATACCGC–AGAGTCTAGA 3′ target**
hsa-miR-17	1568–1590(P/X)	−19.4	151	**miRNA** **3′ UGAUGGAC-GUGACAUUCGUGAAAC 5′** **5′ TCTGCCTGACCTTGT––GCACTTCG 3′ target**
hsa-miR-18a	1514–1535(P/X)	−14.2	144	**miRNA** **3′ AUAGACGUGAUCUACGUGGAAU 5′** **5′ ACCGACCACGGGGCGCACCTCT 3′ target**
hsa-miR-20a	1569–1590(P/X)	−16.7	149	**miRNA** **3′ GAUGGACGUGAUAUUCGUGAAAU 5′** **5′ CTGCCTG–-ACCTTGTGCACTTCG 3′ target**
hsa-miR-20b	1569–1590(P/X)	−19.6	155	**miRNA** **3′ GAUGGACGUGAUACUCGUGAAAC 5′** **5′ CTGCCTG–-ACCTTGTGCACTT CG 3′ target**
hsa-miR-224	281–304(P/X)	−20.3	144	**miRNA** **3′ AUUUGCCUUGGUGAU–CACUGAAC 5′** **5′ TAGGGGGAACTACCGTGTGTCTTG 3′ target**
hsa-miR-483	1726–1744 (X)	−21.4	145	**miRNA** **3′ UCUUCUGCCCUCCUCUCCUCACU 5′** **5′ TAAAGACTGG––––GAGGAGTTG 3′ target**

P, polymerase, S, surface, and X, X open reading frames of HBV.

**Figure 4:**
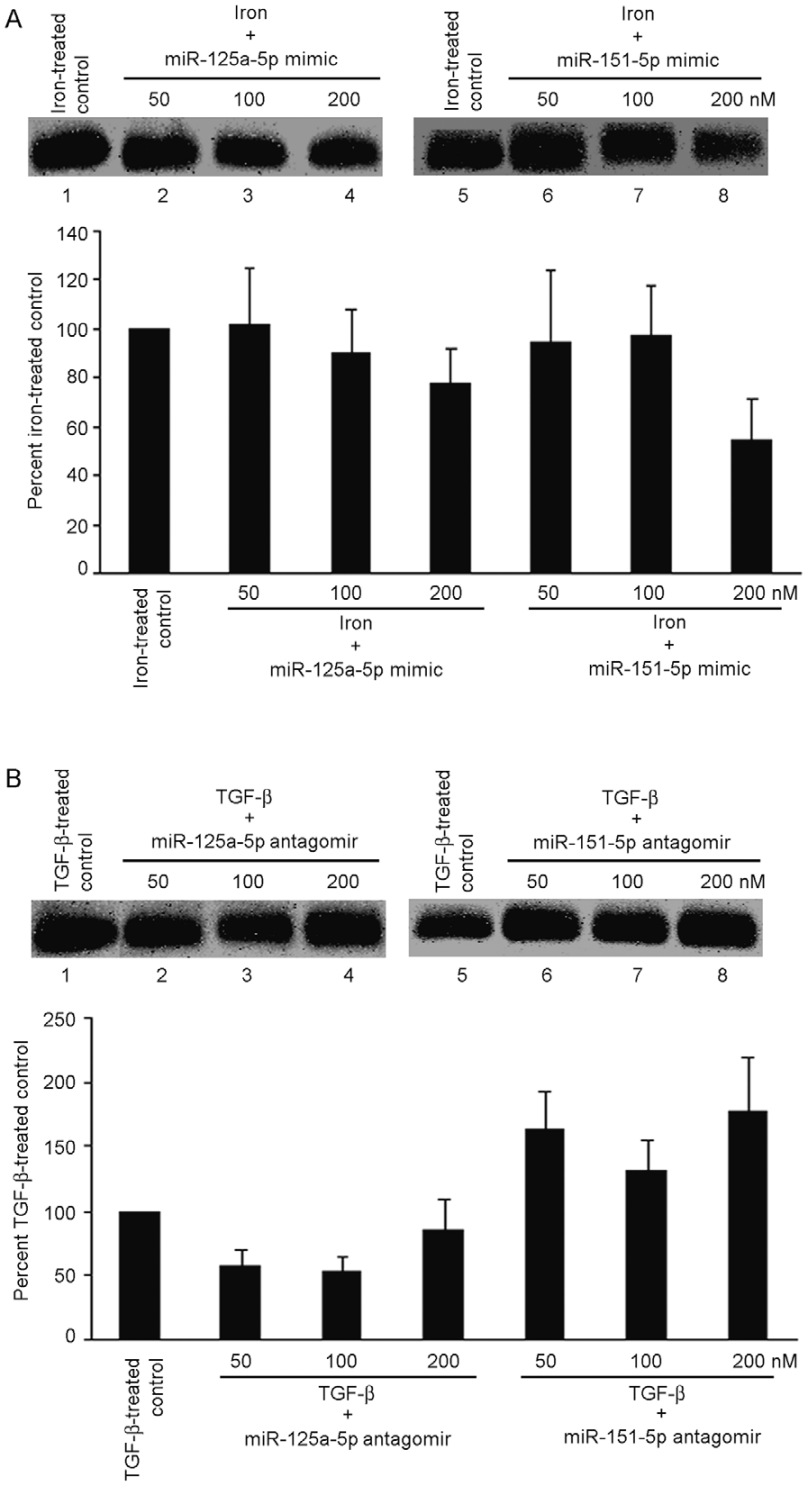
Regulation of HBV replication by agarose gel analysis in cells treated with miRNA. For these studies, 2.2.15 cells were transfected with miRNA mimics or miRNA antagonists followed by administration to transduced cells of either 20 ng/ml TGF-β or 100 µM iron for 48 h. (A) Changes in iron-treated cells are shown after expression of miR-125a-5p or -151-5p mimics. These mimics dose-dependently decreased HBV replication in iron-treated cells. (B) Changes in TGF-β-treated cells are shown after expression of miR-125a-5p and miR-151-5p antagonists. These antagonists dose-dependently reversed TGF-β-induced inhibition of HBV replication. This substantiated the role of intracellular miRNA in regulation of HBV replication by iron or TGF-β.

## Results

### Iron Increased and TGF-β Decreased HBV Replication in 2.2.15 Cells

After initial dose-response studies, we chose to treat cells with 100 µM iron or 20 ng/ml TGF-β for 48 h. In response to TGF-β, cell proliferation decreased slightly with MTT utilization of 87±9% versus 100% in untreated controls, but this was not statistically significant. By contrast, in response to iron, cell proliferation increased slightly with MTT utilization of 118±6% versus 100% in untreated controls, which was also not statistically significant.

We established that 2.2.15 cells showed appropriate responses to iron and TGF-β. In iron-treated cells, hepcidin mRNA expression increased by up to 360-fold, but this was unchanged in TGF-β-treated cells, p<0.05 (**Fig. 1A**). In TGF-β-treated cells, activity of TGF-β receptor serine-threonine kinase increased by up to 4-fold, p<0.05, and was blocked by TGF-β receptor kinase inhibitor (TKI) (**Fig. 1B**). Native agarose gel assays, including southern blotting of these gels, indicated iron increased HBV replication by 20–40% (**Fig. 1C, 1D**). By contrast, TGF-β exerted an effect opposite to iron, since HBV replication decreased by 10–30% in TGF-β-treated cells. Northern and southern analysis confirmed these opposite effects of iron and TGF-β on HBV RNA and DNA expression in 2.2.15 cells (**Fig. 1E, 1F**).

### Transcriptional Changes in TGF-β/BMP Signaling Pathways

In iron-treated 2.2.15 cells, expression of genes in TGF-β/BMP signaling pathways were not different from control untreated cells. However, TGF-β-treated cells showed marked differences in gene expression (**Table 1** **).** We found multiple cytokines in TGF-β superfamily were expressed at higher levels, including left-right determination factor (339-fold) and Nodal (40-fold), which regulate embryonic development. Expression of several cytokines increased, including TGF-β1 (13-fold), TGF-β2 (21-fold), and TGF-β3 (3-fold), BMP1 (5-fold), latent TGF-β-binding proteins (LTBP)-1 (4-fold) and -2 (30-fold), as well as inhibin (INH)-A (11-fold), -BA (5-fold), and -BB (3-fold). Similarly, expression increased of TGF-β and related receptors, including TGF-βR1 (5-fold), TGF-β1-induced transcript 1 (10-fold), BMPR1A (2-fold), BMPR2 (8-fold), and activin receptor 1 (6-fold). Moreover, increased expression was noted of TGF-β transducers, i.e., SMAD-3 (4-fold), SMAD-5 (2-fold), TGF-β-induced (6-fold), and TGF-β-induced factor homeobox 1 (4-fold). Several cell proliferation regulators were expressed more, i.e., Jun (5-fold), JunB (43-fold), Fos (3-fold), p15 (CDKN2B) (26-fold), p21 (CDKN1A) (4-fold), insulin-like growth factor-I (11-fold), and platelet-derived growth factor B (16-fold). Extracellular matrix genes were expressed more, e.g., collagen, type 1, αl (14-fold) and integrin βV (2-fold), which was consistent with fibrogenic-type changes in TGF-β-treated cells. To visualize these gene relationships, we mapped canonical TGF-β/BMP pathways (**Fig. 2**).

We did not determine the effects of TKI on TGF-β-induced gene expression because it successfully blocked TGF-β response in 2.2.15 cells. However, we studied whether TKI would alter cellular responses to iron. In cells treated by iron plus TKI, we found no increases in expression of TGF-β superfamily cytokines or receptors, although expression of Nodal, INHA and LTBP4 declined by 2–3-fold.

### SMAD Protein Expression in Cells

We immunostained cells for phosphorylated SMAD-2, -3 and SMAD-4 proteins to confirm changes in TGF-β/BMP signaling. In untreated cells, SMAD-2 was observed occasionally in cell cytoplasm (**Fig. 3A**). Iron did not alter SMAD-2 expression. This was different in TGF-β-treated cells, since SMAD-2 was now expressed in 82% of cells, p<0.05, along with translocation of the protein in many cells to nuclei. By contrast, iron plus TKI did not affect SMAD-2 expression. SMAD-3 was expressed well under basal conditions and also in iron- or TGF-β-treated cells (**Fig. 3B**). Expression of SMAD-4 changed prominently with significant increases from basal levels in iron- (53%) or TGF-β-treated cells (57%), p<0.05. In cells treated with iron plus TKI, increases in SMAD-4 expression were limited (**Fig. 3C**). These findings indicated that TGF-β signaling contributed to SMAD-4 expression after treatment of cells with either TGF-β or iron. SMAD-4 was largely in cytoplasm under all conditions.

### Iron or TGF-β Altered Cellular miRNA Expression

To identify potential transducers of TGF-β/BMP signaling in HBV replication, we analyzed miRNA expression. We chose to study this mechanism because expression of HBV genes should not have been directly regulated by SMADs activated by TGF-β/BMP signaling. On the other hand, SMADs can regulate expression of cellular miRNA, which, in turn, could have altered HBV replication (see below).

In iron-treated cells, from a total of 814 miRNA, 33 and 36 miRNA were downregulated or upregulated, respectively, versus untreated control cells (**Table 2**). Decreased miRNA expression ranged from Log2 fold of −1.12 (hsa-miR-638) to −10.70 (hsa-miR-20a). Increased miRNA expression ranged from Log2 fold of 1.07 (hsa-miR-23a) to 9.87 (hsa-miR-568). In TGF-β-treated cells, changes were less prominent, with downregulation of 6 and upregulation of 12 miRNA versus untreated control cells. The level of miRNA expression change was also lower, since Log2 fold decreases ranged from −1.25 (hsa-miR-221) to −4.64 (hsa-miR-605) and Log2 fold increases ranged from 1.14 (hsa-miR-23b) to 2.87 (hsa-miR-125a-5p).

Of 6 downregulated miRNA in TGF-β-treated cells, 5 were also downregulated in iron-treated cells: hsa-miR-20b, -605, -638, 663, and -720. Expression of hsa-miR-221was unchanged in iron-treated cells. Expression of 12 miRNA upregulated in TGF-β-treated cells both resembled and differed in iron-treated cells: 6 miRNA were similarly upregulated, hsa-miR-7a, -7d, -7e, -23a, -30c, and -574-5p; 2 miRNA were downregulated, hsa-miR-125a-5p and -21; and expression of 4 miRNA was unchanged, hsa-miR-146a, -23b, -483-5p, and -99b.

The counter-regulation in TGF-β- and iron-treated cells of hsa-miR-125a-5p and -21 was of interest, since these, and other miRNA, may have contributed in HBV replication. RNA hybrid analysis of miRNA listed in **Table 2** identified alignment of 13 miRNA with HBV domains (**Table 3**): hsa-let-7a, -7d, -7e, -106a, -125a-5p, -148a, -151, -17, -18a, -20a, -20b, -224, and -483.

### Regulation of HBV Replication by miRNA Sequences

We selected miR-125a-5p and -151-5p for analyzing their effects on HBV replication. These miRNA are considered to interfere with HBV replication [18], [19]. In response to miR-125a-5p and -151-5p mimics, iron-induced HBV replication declined in dose-dependent fashion by up to 23% and 45%, respectively, p<0.05 (**Fig. 4A**). Expression of miR-125a-5p and -151-5p antagomirs did not affect iron-induced HBV replication. Treatment of cells with antagonists of miR-125a-5p did not reverse TGF-β-induced suppression of HBV replication (**Fig. 4B**). However, antagonist of miR-151-5p reversed TGF-β-induced suppression of HBV replication. Mimics of miR-125a-5p and -151-5p did not affect TGF-β-induced inhibition of HBV replication. This confirmed that individual miRNAs were capable of altering HBV replication.

## Discussion

These studies established 2.2.15 cells were appropriately responsive to iron and TGF-β. In case of iron, this was evidenced by greater hepcidin expression, which is transcriptionally regulated via BMP signaling [20], and requires the cofactors, hemojuvelin and neogenin [21]. Responsiveness to TGF-β was evidenced by receptor kinase activity blocked by TKI. TGF-β changed expression of TGF-β/BMP pathway genes and targets, along with increases in phosphorylated SMAD-2, -3 and -4, as was anticipated. Iron did not alter expression of TGF-β/BMP pathway genes at mRNA levels but SMAD-4 expression linked TGF-β and BMP signaling pathways in iron-treated cells [7]. Despite association of iron with unfavorable outcomes in people with HBV [8], [9], underlying mechanisms directing HBV replication were unknown. Previously, depletion of iron in HepG2 cells decreased HBV production [22]. This was consistent with increased HBV replication here in iron-treated HepG2 cells. We came across another study of TGF-β and HBV replication in HepG2 cells [6], where TGF-β decreased HBV replication, similar to our results. However, underlying mechanisms in how TGF-β directed HBV replication were unknown.

The role of TGF-β-induced SMADs in transcriptional controls was one possibility. Upon ligand-binding, the constitutionally active type II TGF-β receptor phosphorylates type I TGF-β receptor, followed by recruitment of SMAD-2 and -3, which complex with SMAD-4 for transcriptional activation [7]. BMP receptors activate SMAD-1, -5 and -8, which too complex with SMAD-4 for transcriptional activation. Therefore, SMAD-4 connects TGF-β and BMP signaling. Under some situations, TGF-β- or BMP-specific SMADs may be simultaneously activated [23]. We considered that cellular miRNAs could have served intermediary roles in transducing the effects of TGF-β/BMP signaling on HBV replication. Recently, cellular miRNAs attracted much attention as ubiquitous antiviral regulators, including for HBV [18], [19], [24]. miRNAs negatively regulate gene expression by sequence-specific binding of mRNAs followed by mRNA degradation or interference in mRNA translation. The identification of promoter sites in genomic regions encoding miRNAs, including SMAD-binding elements (SBE) [25], [26], was noteworthy to us. Multiple miRNA were shown to contain SBE, e.g., hsa-let-7a, -7b, -7c, -7d, -7e, -7f, -21, -23b, etc. [26]. These miRNA were expressed in iron- or TGF-β-treated cells in our study. However, miRNA expression was different in iron- or TGF-β-treated cells. This difference was not explained by SMADs alone. Also, this difference was not explained by transcription factors regulating iron homeostasis, e.g., hemojuvelin or neogenin, since these are not incriminated in miRNA transcription.

Another explanation was provided by mechanisms in processing of pri-miRNA to mature miRNA [27]. All canonical pri-miRNA undergo sequential cleavages in nucleus and cytoplasm by Dicer or Drosha RNAse III enzymes for maturation. Drosha requires the RNA-binding protein, DiGeorge Critical Region 8 (DGCR8), for pre-miRNA cleavage. Recently, heme was identified as a constituent of Drosha-DGCR8 complex, with the ionic state of heme-bound iron determining whether the complex will be capable of pri-miRNA cleavage [27]. In the ferric state, Drosha-DGCR8-heme complex was highly stable and actively processed pri-miRNA to mature miRNA. By contrast, transition to ferrous state abolished pri-miRNA cleavage by Drosha-DGCR8-heme complex. Thus, alterations in intracellular environment, leading to ferric or ferrous state of iron in Drosha-DGCR8-heme complex, likely accounted for differential miRNA expression in iron- or TGF-β-treated cells.

The nature of miRNA-based host–virus interactions is evolving at present. Several anti-HBV miRNA were previously reported, e.g., hsa-miR-125-5p, -125-b, -151-5p, -199a-3p, -122, etc., including targets in polymerase, core, surface or X genes (15,18,19,28,29). In chronic hepatitis B and HBV-associated HCC, several miRNA were also found in blood, including hsa-miR-7, - 196b, -433, -511, with targets in polymerase or surface genes; hsa-miR-205, targeting X gene; and hsa-miR-345, targeting HBV preC gene [30]. Several of these were present in our list of miRNA expressed differentially in iron- or TGF-β-treated cells, whereas other miRNAs aligning with HBV sequences in our list had previously not been recognized.

Our miRNA expression studies showing modulation of the effects of iron- or TGF-β on HBV replication were instructive. As hsa-miR-125a-5p and -151-5p mimics prevented iron-induced increases in HBV replication, while their antagonists prevented TGF-β-induced decreases in HBV replication, this established miRNA were the principal intracellular effectors of TGF-β/BMP signaling.

Differentially expressed miRNA in iron-treated cells, and for that matter, TGF-β-treated cells, target genes widely, including pathways in cell stress and toxicity, cell proliferation, immune responses, metabolism, etc., which will be relevant for chronic hepatitis. Iron plays major roles in oxidative stress and cytotoxicity, which coupled with perturbations in HBV replication, may alter natural history of hepatitis. TGF-β is especially significant for hepatic growth control and also for fibrosis. Therefore, regulation of miRNA expression by iron and TGF-β will provide opportunities for understanding disease mechanisms in HBV-related liver injury and fibrosis. Moreover, mechanisms directing antiviral miRNA expression in iron- or TGF-β-treated cells should be helpful for drug development, according to considerations discussed previously by other investigators [27].
